# Instruments to Assess Disease-Specific Quality of Life in Dogs: A Scoping Review

**DOI:** 10.3390/ani15121780

**Published:** 2025-06-17

**Authors:** Friederike Felicitas Rhein, Rebecca Klee, Balazs Albrecht, Stephanie Krämer

**Affiliations:** 1 Professorship of Laboratory Animal Science and Animal Welfare & Interdisciplinary Centre for Animal Welfare Research and 3R (ICAR3R), Justus Liebig University Giessen, Frankfurter Str. 95, 35392 Giessen, Germany; stephanie.kraemer@vetmed.uni-giessen.de; 2 Boehringer Ingelheim Vetmedica GmbH, Binger Straße 173, 55216 Ingelheim am Rhein, Germany; rebecca.klee@boehringer-ingelheim.com (R.K.); balazs.albrecht@boehringer-ingelheim.com (B.A.)

**Keywords:** quality of life, well-being, measurement, dogs, instrument, tool, assessment

## Abstract

Quality of life (QoL) assessment has increased in veterinary medicine in recent years, as has the number of attempts to measure it. This review aimed to provide an overview and assess the quality of existing instruments measuring disease-specific QoL in dogs. The PubMed and CAB Abstracts databases were searched in February 2023 using search terms associated with dogs, well-being, and QoL to identify relevant articles which were then evaluated for information on the development and validation processes. For further analysis, 41 publications were selected, of which instruments were available for 30 publications; of these, 24 contained information on item development, while 12 of the 41 instruments described some form of instrument evaluation. Among these 12 instruments, 2 exhibited appropriate test–retest reliability, 7 exhibited acceptable internal consistency, 9 checked at least one face or content validity, and 8 tested at least one sort of hypothesis to contribute to construct validity evaluation. None of the instruments were thoroughly evaluated for all necessary psychometric aspects for their application and result interpretation. Therefore, these instruments’ usage should be carefully considered and subject to restrictions. Further research should focus on establishing guidelines aiming to achieve high standards for instrument development and validation in veterinary medicine.

## Simple Summary

Quality of life is not only important for humans but also for animals. Over the past two decades many efforts have been made to develop questionnaire-based instruments that measure quality of life. Such instruments enable a more objective comparison of quality of life, for example, when evaluating the success of a therapy. Depending on their design, instruments can be either generic—assessing an animal in its whole—or disease-specific, assessing quality of life for a particular disease. We reviewed existing instruments to assess disease-specific quality of life in dogs and compared the development and testing processes with human medicine standards. We found that development processes differed in their developmental approach and were often inadequately described. Furthermore, the reliability and validity of these instruments—meaning whether they truly measure what they are intended to—were often tested only in a few aspects, or not at all. It is important to consider the limitations of an instrument, when using it, to interpret the results correctly. This review highlights the need for more consistent and thorough development of such instruments.

## 1. Introduction

Quality of life (QoL) assessment is a crucial aspect of veterinary practice. As QoL guides decisions regarding treatment as well as euthanasia, it is a key—if not the most important—component of veterinary work [1].

Although the term ‘QoL’ is frequently used, no universally agreed-upon definition vis-à-vis veterinary medicine exists [2]. By contrast, for humans, the World Health Organization defines QoL as an ‘individual’s perception of their position in life in the context of the culture and value systems in which they live and in relation to their goals, expectations, standards and concerns’ [3] (p. 1405).

Despite the lack of a formal definition, the high degree of individuality underscored for QoL among humans also applies to that among animals. The fulfilment of needs and good health play important roles and contribute to a satisfactory QoL; however, QoL transcends these aspects and encompasses all conscious, subjective experiences and feelings [4,5].

In human medicine, QoL assessment is most typically performed through patient self-reporting [5]. However, in the field of veterinary medicine, it is impossible to ask the patient to self-report; therefore, relying on measurements performed by a proxy, such as the treating veterinarian or pet owner, is necessary. Similar challenges in measuring QoL using proxy assessment can be encountered when assessing young children or people with disabilities [4]. Although subjective assessment can be the foundation for variable sources of bias, the incorporation of subjective judgements into the healthcare sector has become a convention—one that has been continuously refined to become increasingly standardised [6] (p. 12).

However, the approaches established in human medicine to quantify QoL differ in form. A fundamental division can be made according to the structure of the tools, some comprising only a single question and others in the form of a questionnaire [7]. Questionnaires can be divided further into ‘generic instruments’, which follow a holistic scope and attempt to assess the individual as a whole, and ‘specific instruments’, which focus on a specific disease, body area, or certain function [7]. Although generic tools offer suitable alternatives to monitor patients in general, they may not reflect subtle changes in specific areas. Therefore, to assess a particular chronic disease’s impact on QoL, employing a disease-specific questionnaire explicitly designed to capture the aspects influenced by the disease can be useful [7].

Developing tools to measure constructs, such as QoL, is a commonplace research phenomenon in psychology, whereby constructs such as depression, anxiety, and happiness can be assessed using psychometric instruments [8]. Generating and selecting items for an instrument is only one component of instrument development. Testing reliability and validity is a crucial step in instrument development, without which, we cannot ensure that the instrument measures what it is supposed to measure. Item generation, selection, and validation of psychometric instruments for humane medicine follow established methods [9,10]. In 2009, the US Food and Drug Administration (FDA) published its requirements for instruments intended to support labelling claims of pharmaceuticals for human medicine [11].

Likewise, in veterinary medicine, various attempts have been made over the past two decades to measure the quality of life among animals. Different tools—both disease-specific and generic, differing in terms of their scope, development process, and validation level—have been developed. To the best of our knowledge, no official veterinary medicine guidelines exist on these tools’ development and validation.

Incorporating a QoL assessment into daily veterinary practice is not only beneficial but also essential as the patient’s QoL is the primary determinant of treatment decisions. Specifically, in treating chronic diseases, where changes are typically extremely minimal and can be overlooked because people have become accustomed to gradual changes in conditions, a standardised survey can be of unparalleled value [1].

Selecting a suitable instrument and assessing its quality is challenging for veterinarians owing to the variety of available tools, inconsistent terminology (e.g., ‘tool’ vs. ‘instrument’ and ‘assessment’ vs. ‘measurement’), and differences in their development and validation approaches. For practitioners, who usually have limited spare time in their daily practice and limited knowledge of psychometric methods, assessing an instrument’s quality is almost impossible.

Although some articles have reviewed existing instruments for dogs [2] and generic instruments for dogs and cats [12], no recent publications have addressed existing disease-specific instruments for dogs.

Therefore, this scoping review intends to provide an overview of the existing structured disease-specific QoL instruments for dogs, including an assessment of their methodology, the quality of item formulation, reliability level, and validation level.

## 2. Materials and Methods

### 2.1. Definition of Terms

No universally agreed definition exists of QoL among animals, which was, therefore, defined in this study per the establishment of a conceptual framework (the process description will be published elsewhere):

*Quality of life in animals is an animal’s individual perception of its current state in relation to its needs, expectations, and desires such that the resulting positive and negative affective states are reflected and observable in the animal’s behaviour and demeanour.*

A QoL assessment instrument or tool was defined as a set of related questions that, collectively, are supposed to provide an overview of the dog’s QoL or well-being. A single scale on ‘How would you rate your dog’s quality of life?’ or a domain of QoL in an instrument designed for another purpose was not considered a tool.

Some concepts—for example, the concept of chronic pain or disease severity—are closely related to the concept of QoL. Measurements of these constructs frequently overlap and are, occasionally, employed interchangeably [13,14]. To obtain a clean separation, a tool was considered a QoL instrument if, in the original development process, the objective was to measure QoL. Therefore, a tool was not considered a QoL instrument if it is used to assess QoL but the original authors developed it as a tool for the measurement of a related construct.

A tool was considered disease-specific if it was designed to be applied to individuals with a specific disease or a disease of a specific organic system or body part.

### 2.2. Search Methods

This scoping literature review was conducted according to the Preferred Reporting Items for Systematic Reviews and Meta-Analyses Extension for Scoping Reviews (PRISMA-ScR) guidelines [15]. The search was conducted in February 2023 for the 2013–2023 period, following Belshaw et al.’s (2015) review [2]. To ensure contingency, the Ovid interface was used to search the CAB Abstracts and Medline (PubMed) databases.

To ensure continuity, the search terms used were those employed in Belshaw et al.’s (2015) review [2]—specifically, dog, dogs, canine, canines, canis, wellbeing, well-being, well being, QoL, quality of life, and quality-of-life, linked with Boolean terms: (dog OR dogs OR canine OR canines OR canis) AND (wellbeing OR well-being OR well being OR quality of life OR QoL OR quality-of-life).

We searched the abstract, title, original title, broad terms, and heading words. The results were exported to Citavi 6.14 (Swiss Academic Software, Wädenswil, Switzerland), and subsequently, the inclusion and exclusion criteria were applied.

### 2.3. Inclusion Criteria

The inclusion criteria were as follows: the publication had to (1) be in English; (2) be published as an original research article in a peer-reviewed journal; (3) address issues concerning domestic dogs; (4) mention QoL/well-being in the title or abstract; (5) describe a questionnaire-based assessment of QoL/well-being in the Materials and Methods section, which would be considered an instrument according to our definitions of terms; and (6) contain a disease-specific instrument.

### 2.4. Exclusion Criteria

The exclusion criteria were as follows: publications that (1) were published in any other language than English; (2) were case reports and reviews published in Congress proceedings or in a non-peer-reviewed journal; (3) addressed any other species than domestic dogs or addressing dogs in a broader context (e.g., rabies situation); (4) did not mention QoL or well-being in the abstract, or title, or clearly did not include them as part of the investigation (e.g., mentioned in sentences such as ‘whether this can improve quality of life has to be further investigated’); (5) did not mention QoL or well-being in the Materials and Methods section, and conducted the evaluation not based on a questionnaire (e.g., laboratory parameters), using a tool that was designed to measure a different construct (e.g., the Helsinki Chronic Pain Index or Canine Brief Pain Inventory) or assessed QoL using a single overall question; and (6) were designed for generic assessment.

### 2.5. Further Selection Process

A single author (FR) applied the inclusion and exclusion criteria, after which the remaining publications contained both those describing an instrument for the first time and those using a previously published instrument. Of the latter group, the original publications were manually searched and included if they satisfied the inclusion criteria.

Additionally, the instruments retrieved from the review by Belshaw et al. (2015) [2] were checked against the inclusion criteria, and those that fulfilled the criteria were included.

**Figure 1:**
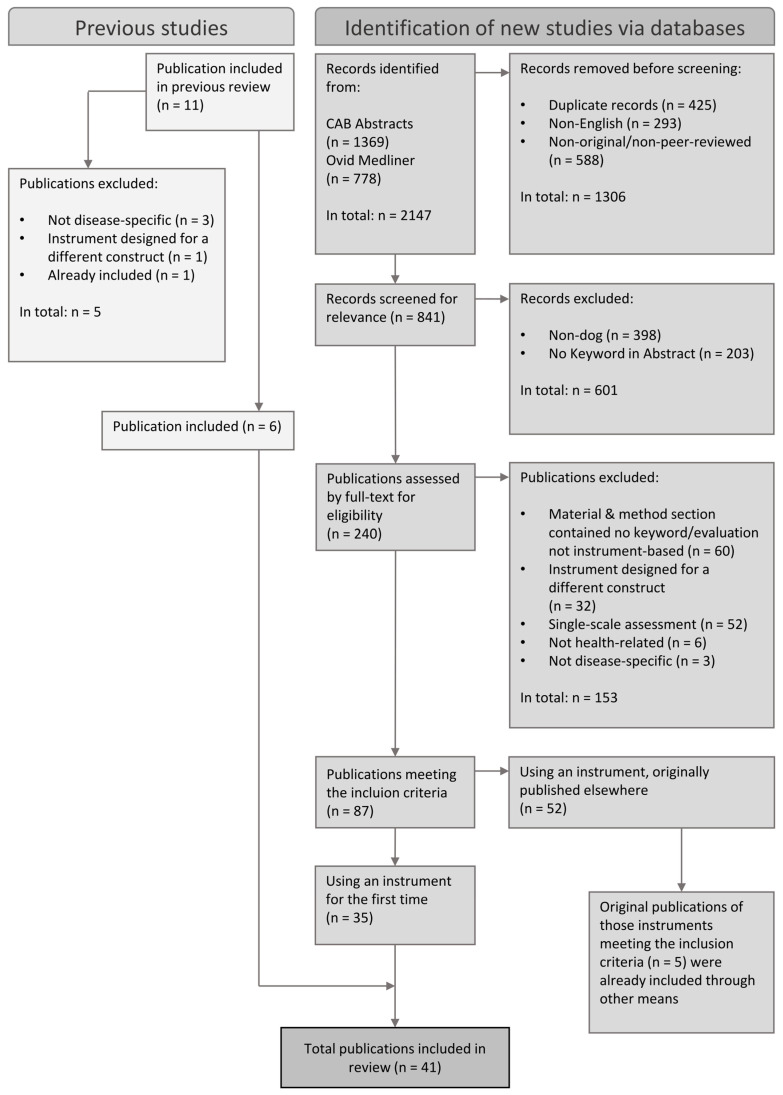
Selection process illustrated in a flow diagram based on the PRISMA 2020 statement.

The resulting publications ultimately formed the sample for further analysis. Figure 1 presents the selection process illustrated as a PRISMA 2020 flow diagram.

### 2.6. Data Collection

The retrieved publications were screened for the following: (1) disease specification and whether the intended construct measured was QoL or well-being; (2) availability of the complete instrument; (3) information regarding the process of item generation and/or selection, based on the criteria outlined in Table 1; and (4) information regarding any testing or evaluation of the instrument, as described in Table 2.

Publications for which a complete instrument was available were screened for the characteristics described in Table 3.

### 2.7. Extraction of Disease Specification and Construct Measured

Information regarding the disease, condition, organ system, or body region for which the instrument was designed was extracted. Owing to the search terms used, instruments measuring QoL and instruments measuring well-being were retrieved. Therefore, information was also extracted based on whether the construct that was intended to be measured was QoL or well-being.

### 2.8. Extraction of Instrument Availability

Practitioners and clinicians in need of an instrument benefit from easy access to copies of the questionnaire. Therefore, the study assessed whether a copy of the instrument was included in the publication itself, the appendix, or the supplementary material, or whether the authors would have to be contacted to obtain a copy.

### 2.9. Evaluation of the Item Generation and/or Selection Process and Any Form of Evaluation and/or Testing

The criteria for assessing the process of instrument design and validation were based on the criteria established by the US FDA [11], a scientific committee report on developing disease-specific QoL [10], the Consensus-based Standards for the Selection of Health Status Measurement Instruments (COSMIN) risk of bias checklist, and COSMIN guidelines for systematic reviews of patient-reported outcome measures [17,18,19,20] that were adapted to veterinary medicine.

## 3. Results

The initial search returned 2147 publications, of which 425 were duplicates and were removed. Of the 1722 remaining publications, 87 fulfilled the inclusion criteria. Figure 1 presents the application of the inclusion and exclusion criteria.

Publications that contained an instrument used for the first time (n = 35), including modifications used for the first time, were employed for further analyses. Additionally, Belshaw et al.’s (2015) review [2] was checked for relevant publications (Figure 1). For publications in which a previously published tool was used (n = 52), the original publications were searched, and it was confirmed whether they fulfilled the inclusion criteria. The original publications that fulfilled the inclusion criteria (n = 5) had already been included through other means. Finally, 41 publications were included for further examination.

### 3.1. Disease Specification and Construct Measured

The instruments addressed different types of disease grouped by affected organs or organic systems (Table 4). Owing to the heterogeneous appearance of cancer, the variety of instruments used to address cancer are presented in Table 5.

For two instruments, well-being was the objective of the measurement; for 39 instruments, the measurement’s objective was QoL.

### 3.2. Instrument Availability

For 30 publications, the questionnaires used were fully available, integrated into the publication itself, or copies could be found in the appendix or supplementary material. Five publications did not contain the questionnaire itself but displayed the results of the items in a table; thus, an overall impression of the instrument could be obtained. Two publications stated that copies of the questionnaire were available from the authors upon request. Four publications did not contain either the questionnaire, a display of the items, or an availability statement.

### 3.3. Information About Item Generation and/or Selection

Sixteen publications did not provide any information on the process of devising the items; they used only a set of questions without explaining why they considered them suitable or where they derived them from. Twenty-four publications contained information on the development of the items (to various extents). One publication could not be classified owing to unclear references.

Five publications explicitly reviewed the literature as part of the instrument development process. Fourteen publications derived their items from existing questionnaires, but only two commented on their selection criteria or why they modified a particular instrument rather than using the originally published version.

Eight instruments were influenced by human medicine in different ways. They reflected that the items regarding content, question formatting, or domain structure were derived from a corresponding questionnaire in human medicine; however, discussions were also conducted with people affected by the disease to inform the process of item generation.

In 10 publications, the dogs’ owners could influence the instrument’s design. In three publications, they were used as a source to generate items through qualitative interviews; in two publications, they could exert influence by adapting the items during the application of the instrument; and in five publications, they were part of the testing process in different ways, for example, through pre-testing or in evaluating readability, content validity, face validity, or the instrument in general.

### 3.4. Information About Instrument Testing

Of the 24 publications that described the development of items, 10 also described some form of testing. One publication mentioned that validation had occurred, but no further details were provided.

Of the 16 publications in which the process of item generation and selection was not described, two contained more detailed information on the validation. In another publication, validation was mentioned; however, the data were not presented.

Twelve instruments described some form of instrument evaluation. Table 6 presents a detailed assessment of the aspects tested.

### 3.5. Characteristics of the Available Instruments

Among the publications that reported testing, the instruments were available in 11 of them. Six of them were named. The number of items ranged from 5 to 24, and the scaling of the items varied across the instruments. Visual analogue scales (VAS), numerical rating scales (NRS), adjectival scales, and 5-point Likert scales were used. Adjectival scales had values assigned so an overall score could be calculated. The overall scores were calculated for 7 of the 11 instruments. In three cases, the item scores were summed; one overall score was based on calculating the percentage of the maximal possible score, and another was based on calculating the average item score. Only one instrument had a weighted score. The characteristics are presented in Table 7.

## 4. Discussion

This review provides an overview of the disease-specific QoL instruments currently available for use in dogs and, therefore, updates the one from 10 years ago [2]. After the review identified 2147 records, inclusion and exclusion criteria were applied, and finally, 41 publications were included for further analysis. The field of application differed greatly, with more than a quarter of the instruments designed for use in cancer. As cancer treatment is accompanied by severe side effects and is often palliative, careful monitoring of QoL is essential to constantly weigh costs and benefits as well as to release the dog from its suffering in a timely fashion. Furthermore, the European Medicines Agency recommends QoL as an endpoint in clinical field studies for the authorisation of anticancer medicinal products for dogs and cats [34].

For 30 of the 41 publications, the instrument used was fully available either within the publication itself or in the appendix or supplementary material; in the remaining publications, the instruments were not made available. The unavailability of instruments makes their use less likely, as the author must be contacted first to receive the instrument. Additionally, numerous authors may be unavailable or may not respond, and the instrument, if provided, may turn out not to fit its intended purpose. Therefore, it is unlikely that practitioners will take the time to contact an author in a busy practice where the instrument is normally needed immediately. Therefore, no authors were contacted for this study, as it was intended to provide an overview that simplifies the choice of an instrument for daily practice.

Sixteen of the 41 publications did not provide any information on the development process. Additionally, for 14 of the 41 publications, questions were derived from existing questionnaires, but 12 of these did not provide any information about the item selection process. The process of devising or selecting the items ‘is far from a trivial task, since no amount of statistical manipulation after the fact can compensate for poorly chosen questions; those that are badly worded, ambiguous, irrelevant, or—even worse—not present’ [9] (p. 19). Therefore, the process of devising the items provides clues about the quality of an instrument, and furthermore, the FDA recommends that the process of item generation and selection should be documented in a comprehensible way [11].

The involvement of patients in the target population during the process of item development is considered crucial in human medicine [10,11]. However, in veterinary medicine, we obviously cannot ask the patients themselves; therefore, owners and veterinarians must serve as a proxy. Accordingly, those who will apply the questionnaire later (usually the owners) should be involved in the process. Owners were included in the process of item generation for only three instruments [24,28,29], with the authors of one article emphasising the input of owners as the most valuable source and suggesting the inclusion of owner input for every owner-reported measurement tool to be developed [24]. As has been reported previously, owners are perfectly capable of describing behavioural changes [35].

However, carefully devising and selecting items is only part of the equation for producing high-quality instruments. Testing reliability and validity and, if necessary, improving the items, formatting, or structure of the instrument, if they are insufficient, is the other major part of instrument development. Twelve of the 41 publications described some form of reliability and/or validity. The degree of testing differed widely, and none of the instruments were tested for every aspect. These findings were consistent with those of Belshaw (2015) [2], Doit (2021) [36], and Fulmer (2022) [12].

Various aspects of reliability can be assessed. Test–retest reliability was assessed for 3 of the 12 publications with appropriate statistical tests. Two publications [24,29] showed good test–retest reliability (≥0.70), which is considered sufficient for good reliability [17]. Inter-rater reliability was assessed for only one instrument [29]. If inter-rater reliability has not been tested or is insufficient, it cannot be guaranteed that administrations carried out by different individuals are comparable. Therefore, if inter-rater reliability has not been established and multiple administrations on the same animal are planned, a questionnaire should be administered by the same person each time to avoid unreliable results.

High internal consistency contributes to acceptable reliability, but internal consistency alone is insufficient as evidence of reliability for clinical trial purposes when test–retest reliability is not assessed [11]. Internal consistency was analysed and confirmed to be acceptable (Cronbach’s alpha ≥ 0.70) for 7 of the 12 publications. However, one publication was missing the calculation details [27], and another publication [28] did not calculate the internal consistency separately for their dog QoL instrument but included an owners’ QoL instrument in their calculation. Strictly speaking, internal consistency should only be assessed if the unidimensionality of a scale or subscale was evaluated. Therefore, calculations for instruments for which unidimensionality has not been checked or for which two constructs have been combined should be treated with caution.

Principal component analysis (PCA) or exploratory factor analysis (FA) can be performed to find an underlying structure. PCA was conducted for 3 of the 12 publications, and FA was conducted for 1 of the 12 publications. Assessing the underlying structure can facilitate omitting unnecessary items that do not contribute to the measurement. If unnecessary items are omitted, the length of a questionnaire decreases, and the respondent burden can, therefore, be lowered [10]. For reflective measurement models in which the items are supposed to be correlated, an assessment of the underlying structure should be conducted [17].

A test’s validity describes its ability to measure what it is intended to measure [6] (p. 30). Content validity must be prioritised [11]. This determines whether every relevant aspect is covered and whether no unnecessary aspects that could increase the measurement error are included. ‘Face validity’, which is sometimes used similarly to the expression ‘content validity’, but has a slightly different meaning, describes whether an instrument appears to be measuring what it is supposed to measure [10].

Content validity was evaluated for 7 of the 12, and face validity in 4 of the 12 publications. In two cases, both content validity and face validity were evaluated, leaving three questionnaires untested for their content. Content and face validity were most frequently established through informal discussions or feedback from the pilot questionnaire, with a few authors not even using the terms ‘content validity’ or ‘face validity’ [26,28,29,31], while others established content and face validity through a comprehensive approach, for example, through semi-structured interviews [24] or focus group discussions [30]. As all 12 questionnaires were owner-reported QoL assessments, owners should not only be included in the development process, as discussed above, but also in content validation.

Testing for correlations with measurements of closely related constructs, such as disease severity, evaluates convergent validity and is, therefore, part of hypothesis testing, which contributes to the establishment of construct validity. This testing was performed for 3 of the 12 publications. Correlations with related constructs should be between 0.30 and 0.50 [17]. Correlations below 0.30 would indicate that either one of the measurements or the hypothesis that the measurements are related is incorrect [9] (p. 240). Correlations that are too high would indicate that the same construct had been measured and that the instrument is just a new way to measure the construct that the other instrument measures [9] (p. 240). The correlations ranged from being too low or barely acceptable [28] to reflecting slightly too high [23] to very high [30] correlations.

Another method for evaluating construct validity was to compare the questionnaire results with an overall score, which was performed for 4 of the 12 publications. Correlations with measurements of the same construct should correlate highly, at least ≥0.5 [17], which was the case in all but one [29] of the instruments.

Comparing ‘extreme groups’, namely, groups that are expected to score very differently in the examined construct, is a third way of contributing to construct validity [11,16]. This was conducted for 7 of the 12 publications, and significant (*p* < 0.05) differences between the groups could be observed in the tested instruments. The exception was the instrument in Marchetti et al. (2021) [27], which comprised five domains that were tested separately, only four of which showed a significant difference. However, as statistical significance also depends on the sample size and large sample sizes can make minimal differences statistically significant, the magnitude of correlations or differences should be analysed [37]. Two studies [28,32] used analysis of variance (ANOVA) as the statistical test without prior testing for normal distribution—a requirement for the valid use of this statistical test.

The instruments differed widely in terms of the number of items (5–24). Generally, as many items as necessary should be used to cover every aspect and guarantee content validity, and as few items as possible to minimise the respondent burden [10]. Schofield et al. (2019) [29] were the only authors to report the time it took owners to complete the questionnaire. As the 19 items took six minutes to complete, it can be assumed that the other instruments were also of a reasonable length, making them easy to complete while the owner waited.

In summary, many instruments described their development process inadequately, did not test at all, tested only a few aspects, or modified an existing questionnaire without reassessing its reliability and validity. Furthermore, the statistical methods used had some limitations. One possible explanation for this phenomenon is that the instruments were developed by people who believe that such an instrument is needed and try to develop it but are unaware of the large body of research conducted on instrument development in the psychological field and, therefore, with the best intentions, believe that ‘a few questions will do the job’. In addition, even in human medicine, uncertainties remain regarding the taxonomy and evaluation of measurement properties, which, in turn, leads to uncertainties when assessing the quality of a patient-reported outcome measure [38,39]. Therefore, it is not surprising that veterinary medicine faces similar challenges.

This study has several limitations. The search terms used were the same as those used by Belshaw et al. (2015) [2]. It cannot be guaranteed that no additional instruments would have been found if other or additional terms, such as ‘welfare’, had been included. However, this review focused specifically on instruments from the health sector in which the terms ‘QoL’ or ‘health-related quality of life’ (HRQoL) are used most frequently, with well-being often incorporated in the definitions of QoL or HRQoL [40]. Among the 41 extracted publications related to disease-specific evaluations, only 2 had well-being as the target construct. Among the 11 instruments described in more detail, the measured construct was only QoL, which supports the hypothesis that the most commonly used health-related term is quality of life.

As the instruments were frequently modified, clearly classifying them as distinct instruments was challenging. To ensure accuracy, each set of questions was included as soon as any changes were made. To avoid confusion, the use of the term ‘instrument’ should be reconsidered and should only be applied in future to sets of questions that have undergone testing and demonstrated adequate psychometric properties.

For future research, it is recommended to collaborate with researchers from other disciplines to incorporate as much knowledge as possible to advance research on QoL measurement in veterinary medicine that is in the best interests of animals. Developing guiding information similar to the COSMIN guidelines [17,18,19,41,42] for veterinary medicine would also be beneficial.

## 5. Conclusions

Although research on QoL measurement is underway, we are still far from establishing rules for developing and testing psychometric instruments; therefore, sufficiently tested instruments in veterinary medicine are lacking. Nevertheless, if we understand the individual shortcomings of each tool, they can still be used to facilitate certain processes in daily practice. The overview provided in this review helps practitioners decide on an instrument and highlights the untested areas of the instruments, so they can be used responsibly and subject to the restrictions.

Future research should focus on establishing common guidelines for the development and testing of psychometric instruments, such as QoL instruments, in veterinary medicine. Additionally, the instruments discussed in this review should be reassessed to properly evaluate their psychometric properties and, if necessary, refine them to make them widely usable. If such a reassessment is undertaken, it should be based on established methodologies and guidelines from human medicine and psychology, where psychometric testing has a longer tradition and well-defined standards.

Overall, the increasing focus on considering QoL is commendable. Subsequently, the focus must be on establishing a sound and comprehensive research foundation to build upon.

## Figures and Tables

**Table 1:** Criteria for assessing the process of item generation and/or selection.

Criteria
Was the instrument based on/adapted from previously published veterinary questionnaires?
If so, were there any clues about the motivation for modification or of the criteria for item selection?
Was there any orientation towards humane medicine?
Was a literature review used to generate items?
Were experts involved in the development of the items?

**Table 2:** Criteria to assess the psychometric properties of the instruments.

Property	Aspects	Description
Comprehensibility		An attempt has been made to ensure that the instrument is understandable by the target group, e.g., qualitative focus group discussions, cognitive interviews with the target group, or quantitative assessments for clarity.
Reliability		The reliability of a test indicates that the measurement of a test is subject to minimal errors, and results are independent of influences other than the object being measured itself [9] (p. 159).
	Test–retest reliability resp. intra-rater reliability	The ability of the instrument to produce repeatable results if the same person administers the tool to the same individual at two or more time points; the correlation should be calculated using the ICC * [10] or Cohen’s kappa when no underlying continuum exists [9] (pp. 174–178).
	Inter-rater reliability	The reproducibility of a score when different people administer the instrument to the same individual. Calculation of the correlation should be performed using the ICC * [10] or Cohen’s kappa if no underlying continuum exists [9] (pp. 174–178).
	Internal consistency	The correlation degree between the individual items within a questionnaire. High internal consistency contributes to good reliability. It should be calculated using Cronbach’s alpha [9] (p. 88).
Validity ^a^		A test’s validity describes the test’s ability to measure what the test was supposed to measure [6] (p. 30).
	Content validity	The degree to which an instrument adequately incorporates every relevant aspect and leaves out unimportant aspects. Content validity should be established over every other aspect of validity. It can be established based on the judgement of experts [9] (p. 233), or when it is about an instrument measuring an outcome related to the subjective experience of patients. It can also be established through the input of patients given during the development process or via interviews wherein patients are asked whether the instrument encompasses all important aspects [11].
	Face validity	The degree to which the instrument appears to measure what it intends to measure. It may increase the acceptance of people who are supposed to use it or lead respondents to consciously or subconsciously adjust their answers to satisfy expectations [9] (p. 79) [10].
	Construct validity —hypothesis testing.	The degree to which an instrument measures previously formulated hypotheses: *Known-groups comparison* Comparison of two populations known to be different regarding the construct being measured, e.g., sick and healthy individuals [11,16] *Comparison with a separately asked overall assessment* Comparing a multi-item measure with a single-item question about the construct in general, where the results are expected to correlate. *Convergent validity* Testing how the evaluated construct behaves in relation to measurements of other constructs known to be related. Convergent validity describes that the measurements of two supposedly related constructs should behave similarly [6] (p. 34), e.g., the results of a QoL measurement are expected to correlate with the scores of an instrument to measure disease severity, hypothesising that disease severity influences QoL.
	Construct validity—Structural validity/unidimensionality.	Statistical assessment of the underlying structure; principal component or factor analysis to identify the underlying dimensions, used to reduce the number of variables, aids in omitting redundant items, of which the underlying driving component is already sufficiently represented elsewhere; latent variables to discover sub-concepts of a construct and their relations.
Responsiveness		Information has been provided on how responsive the tool is to changes in condition.
Interpretability		Information has been provided on the meaning of a change in the score and a report of the minimal important difference.

* ICC = Intraclass correlation coefficient. ^a^ Criterion validity, which is the degree to which the measurements of an instrument meet an externally applied criterion (‘gold standard’), was not assessed, as no gold standard exists for QoL measurement in veterinary medicine.

**Table 3:** Characteristics for which the fully available instruments were screened and compared.

Name	Score Calculation	Domains	Items
Was the instrument named?	Was there an overall score?	Were the items grouped into domains?	How many items were contained?
	If yes, how was it created?	If yes, how many were there?	How were the items scaled?
	If yes, was there a weighted score?		

**Table 4:** Instruments categorised by type of disease addressed.

Disease Type	Number of Instruments
Cancer	11
Cardiac disease	7
Orthopaedic disease/condition	7
Neurological disease	3
Portosystemic shunt	3
Skin disease	3
Respiratory disease	2
Enteric disease	2
Haematological disease	1
Obesity	1
Metabolic disease	1

**Table 5:** Variety of body regions and organic systems addressed by instruments developed for QoL in patients with cancer.

Specific Focus	Number of Instruments
Cancer in general	2
Receiving chemotherapy	1
Appendicular osteosarcoma	1
Nasal carcinoma	1
Urinary bladder carcinoma	1
Spontaneous melanoma	1
Lung carcinoma	1
Oral malignant melanoma	1
Mast cell tumour	1
Pain secondary to cancer	1

**Table 6:** Assessment of the testing of 12 instruments where further details about the testing process were provided.

		Budke et al., 2008 [21]	Favrot et al., 2010 [22]	Freeman et al., 2005 [23]	Giuffrida et al., 2018 [24]	Iliopoulou et al., 2013 [25]	Lynch et al., 2011 [26]	Marchetti et al., 2021 [27]	Noli et al., 2011 [28]	Schofield et al., 2019 [29]	Weiske et al., 2020 [30]	**Wessmann et al., 2014** [31]	**Yazbek and Fantoni, 2005** [32]
	Instrument available?	Y	Y	Y	Y	N	Y	Y	Y	Y	Y	Y	Y
	Development described?	Y	Y	Y	Y	Y	Y	N	Y	Y	Y	Y	N
	Comprehensibility?	N	N	Y	Y	N	Y	N	Y	Y	Y	N	Y
Reliability	Test–retest reliability/ Intra-rater reliability?	N	N	(Y) ^c^	Y	N	N	N	N ^g^	Y	Y ^j^	N	N
	Inter-rater reliability?	N	N	N	N	N	N	N	N	Y	N	N	N
	Internal consistency?	na ^a^	N	Y	Y	N	N	Y ^f^	(Y) ^h^	Y	Y	Y	N
Underlying structure	Statistical assessment of the underlying structure?	na ^a^	N	N	Y	N	N	N	N	Y	Y	Y	N
	Unidimensionality/ principal component analysis				N					Y	Y	Y	
	Structural validity/ factor analysis				Y					N	N	N	
Validity	Face validity	Y	N	Y	N	N	Y	N	N	N	Y	N	N
	Content validity	na ^a^	Y	N	Y	N	Y	N	Y	Y	Y	Y	N
	Construct validity—hypothesis testing *known-groups comparison*	Y	N	N	N	Y ^e^	N	Y	Y	Y	Y	N	Y
	Construct validity—hypothesis testing *comparison instrument overall score with a single-question overall assessment*	Y	na *	Y	N	N	na *	na *	N	Y	Y	na *	N
	Construct validity—hypothesis testing *convergent validity with related constructs*	N	N ^b^	Y ^d^	N	N	N	N	Y ^i^	N	Y	N ^k^	N
	Responsiveness?	N	N	N	Y	N	N		N	N	N	N	N
	Interpretability?	N	N	N	N	N	N		N	N	N	N	N

N, no; Y, yes; na, not applicable. * No overall instrument scores (see also Table 7). ^a^ In the instrument developed by Budke et al. (2008) [21], owners selected the items themselves; therefore, internal consistency, statistical assessment of the structure, and content validity could not be assessed. ^b^ Instrument containing one overall assessment. Only the overall assessment was compared to the disease severity measurements and not the items of the instrument itself. ^c^ Spearman’s rank correlation was used to assess test–retest reliability. Spearman’s rank correlation should only be deemed adequate if proof exists that no systematic change has occurred [17,18,19]. ^d^ Freeman et al. (2005) [23] reported a correlation with the International Small Animal Cardiac Health Council (ISACHC) classification as criterion validity. Because the instrument aims not to measure disease severity but QoL; in this overview, it is considered to represent convergent validity, which is a part of construct validity. ^e^ Each dog served as its own control, as the owners were asked about the animal before diagnosis when the dogs were healthy. However, retrospective questioning must be handled carefully, given the possibility of recall bias [33]. ^f^ Cronbach’s alpha was reported, but no details were provided regarding the reliability testing process. ^g^ The test–retest reliability was calculated using Cronbach’s alpha, a measure of internal consistency. ^h^ Internal consistency was not calculated separately for the dog QoL instrument but included an instrument for owners’ QoL in their calculation. ^i^ Noli et al. (2011) [28] reported the correlations between their instrument and the CADESI-03 severity scale, and between their instrument and a VAS pruritus assessment, as criterion validity. As the instrument is designed to measure QoL, rather than disease severity, in this overview, the correlations are considered to represent convergent validity, which is part of construct validity. ^j^ The intraclass correlation coefficients ranged between 0.17 and 0.57, which suggests a low-to-moderate correlation. For good test–retest reliability, the ICC should be ≥0.70 [17]. ^k^ The authors stated that correlations supporting construct validity had been confirmed, but they described no hypothesis testing in the Materials and Methods section and provided no results.

**Table 7:** Characteristics of the instruments.

	Instrument Named? If Yes, How?	Field of Application	Number of Items	Item Scaling	Divided into Domains?	If Yes, How Many?	Overall Score Calculated?	Weighted Score?	Calculation Method
Budke et al. (2008) [21]	No	Spinal cord injuries	5	VAS	No		Yes	Yes	Multiplying the weight percentage by the VAS score
Favrot et al. (2010) [22]	No	Atopic dermatitis	14	5-Point Likert	No		No	No	
Freeman et al. (2005) [23]	Functional evaluation of cardiac health (FETCH)	Heart disease		0–5 NRS	No		Yes	No	Item scores summarised
Giuffrida et al. (2018) [24]	Canine Owner-Reported Quality of Life (CORQ)	Cancer	17 ^a^	0–7 NRS	Yes	3	Yes	No	The sum of item scores divided by the total number of items
Lynch et al. (2011) [26]	Cancer Treatment Form	Cancer	22 ^a^	1–5 NRS + one VAS	Yes	8	ND		
Marchetti et al. (2021) [27]	No	Chronic inflammatory enteropathy	5	1–10 NRS	No		No		
Noli et al. (2011) [28]	No	Skin diseases	7	4-point adjectival	No		Yes	No	Item scores summarised
Schofield et al. (2019) [29]	CushQol-pet	Cushing’s syndrome	19	4-point adjectival	No		Yes	No	The sum of item scores divided by the maximum possible score
Weiske et al. (2020) [30]	CanBrainQOL-24	Intracranial diseases	24	5-point adjectival ^b^	Yes	3	Yes	No	Item scores summarised
Wessmann et al. (2014) [31]	Epilepsy disease-specific quality of life list of key questions (EpiQol)	Idiopathic epilepsy	16 ^c^	5-point Likert	Yes ^c^	2 ^c^	No		
Yazbek and Fantoni (2005) [32]	No	Pain secondary to cancer	12	4-point adjectival scale	No		Yes	No	Item scores summarised

ND, not described. ^a^ Plus one overall QoL assessment on a VAS. ^b^ The authors called it a Likert scale, but it is an adjectival scale because it is unipolar [9] (p. 44). ^c^ The EpiQol contains 36 questions in seven domains, but only five domains (containing 20 questions) relate to caregiver burden and owner QoL.

## Data Availability

No new data were created or analysed in this study. Data sharing is not applicable to this article.

## Abbreviations

The following abbreviations are used in this manuscript:

QoL	Quality of Life
PRISMA	Preferred Reporting Items for Systematic Reviews and Meta-Analyses
PRISMA-ScR	Preferred Reporting Items for Systematic Reviews and Meta-Analyses Extension for Scoping Reviews
FDA	US Food and Drug Administration
COSMIN	Consensus-based Standards for the Selection of Health Status Measurement Instruments
ICC	Intraclass Correlation Coefficient
VAS	Visual Analogue Scale
NRS	Numerical Rating Scale
PCA	Principal Component Analysis
FA	Exploratory Factor Analysis
HRQoL	Health-related Quality of Life
